# Neural Activity Is Dynamically Modulated by Memory Load During the Maintenance of Spatial Objects

**DOI:** 10.3389/fpsyg.2018.01071

**Published:** 2018-07-03

**Authors:** Yali Pan, Zheng Tan, Zhiyao Gao, Yanyan Li, Liang Wang

**Affiliations:** ^1^CAS Key Laboratory of Mental Health, Institute of Psychology, Beijing, China; ^2^Department of Psychology, University of Chinese Academy of Sciences, Beijing, China; ^3^CAS Center for Excellence in Brain Science and Intelligence Technology, Shanghai, China

**Keywords:** visuospatial working memory, memory load, dynamic neural activity, alpha oscillation, scalp EEG

## Abstract

Visuospatial working memory (WM) is a fundamental but severely limited ability to temporarily remember selected stimuli. Several studies have investigated the underlying neural mechanisms of maintaining various visuospatial stimuli simultaneously (i.e., WM load, the number of representations that need to be maintained in WM). However, two confounding factors, namely verbal representation and encoding load (the number of items that need to be encoded into WM), have not been well controlled in previous studies. In this study, we developed a novel delayed-match-to-sample task (DMST) controlling for these two confounding factors and recorded scalp EEG signals during the task. We found that behavioral performance deteriorated severely as memory load increased. Neural activity was modulated by WM load in a dynamic manner. Specifically, higher memory load induced stronger amplitude in occipital and central channel-clusters during the early delay period, while the inverse trend was observed in central and frontal channel-clusters during late delay. In addition, the same inverse memory load effect, that was lower memory load induced stronger amplitude, was observed in occipital channel-cluster alpha power during late delay. Finally, significant correlations between neural activity and individual reaction time showed a role of late-delay central and frontal channel-cluster amplitude in predicting behavioral performance. Because the occipital cortex is important for visual information maintenance, the decrease in alpha oscillation was consistent with the cognitive role that is “gating by inhibition.” Together, our results from a well-controlled DMST suggest that WM load not exerted constant but dynamic effect on neural activity during maintenance of visuospatial objects.

## Introduction

When immersed in a cluttered environment filled with objects, the limited mind can only remember items perceived to be relevant for later behavioral purposes. This cognitive ability is known as working memory (WM), which consists of verbal and visual forms (Baddeley, 2012). Although verbal WM has been studied as a process by which several verbal stimuli are held as a chunk in the mind (Miller, 1956), the study of multi-object visual WM has elicited some controversy.

One key issue of visual WM is how the brain maintains stimuli in the absence of visual objects. In early times, many researchers used the N-back task to determine the underlying mechanism (Cohen et al., 1997; Gevins et al., 1997). However, this task confuses the three stages of visual WM, namely encoding, maintenance, and retrieval. Researchers then turned to the Sternberg task (Raghavachari et al., 2001; Jensen and Tesche, 2002; Jensen et al., 2002; Howard et al., 2003; Tuladhar et al., 2007) (for an oscillatory model of Sternberg task, see Jensen and Lisman, 1998). In the classical Sternberg task (Sternberg, 1966), the stimuli are consonants presented sequentially. At the very beginning of the task, stimuli are encoded in the visual form and then transformed quickly into verbal representations, in which stimuli can be maintained with the lowest amount of effort (Posner et al., 1969). According to Baddeley’s three components model of WM (the central executive, visuospatial sketchpad, and phonological loop) (Baddeley, 1992), verbal and visual representations are supported by different components and may recruit distinct neural circuits. Therefore, verbal representation during maintenance is a major confounding factor in the study of visual WM. Additionally, sequentially presented stimuli will inevitably confound the memory load effect with recency and temporal memory effects. Furthermore, presenting stimuli in this manner provides limited information about spatial locations. In the delayed-match-to-sample task (DMST), stimuli are presented simultaneously, suitable for investigating visuospatial WM, and the different stages of the process are clearly separated. Typically, WM load is indicated by the number of representations that need to be maintained during delay period. While in this kind of paradigm, it means the number of stimuli displayed in sample (Luck and Vogel, 1997; Todd and Marois, 2004; Palva et al., 2011; Roux et al., 2012), which confuses with the encoding load (the number of items that need to be encoded into WM). Therefore, increasing WM load by adding more items in sample display will inevitably induce greater encoding load. Encoding load is another confounding factor that needs to be controlled, especially when investigating the time course of WM load effects on representation maintenance, since the exact end time of sample encoding is not known.

In this study, we modified the classical DMST, with all spatially dots (non-colorized and colorized dots) presenting simultaneously during the sample display. However, only the number of colorized dots, rather than the total number of dots, comprised the memory load level. Therefore, the encoding load (i.e., total number of dots in sample display) was constant across different memory load levels. In addition, we employed a verbal WM task embedded in the DMST as a control so that verbal representations would be fully occupied by the verbal task, ensuring pure visuospatial representations for visuospatial stimuli.

Previous studies suggested that WM representations are maintained by sustained activity throughout the retention interval (Braver et al., 1997; Tallon-Baudry et al., 1999; Raghavachari et al., 2001; Jensen and Tesche, 2002; Onton et al., 2005; Palva et al., 2010) (for a review, see Leavitt et al., 2017). However, accumulating evidence indicates that neural activity in the delay period is not sustained but dynamically changed (Bashivan et al., 2014; Leszczynski et al., 2015; Siebenhuhner et al., 2016). This idea is demonstrated by the “activity-silent model” (Stokes, 2015). However, the classic paradigms in this model involve making one specific WM representation task irrelevant and putting it into the activity-silent state based on an instruction or cue. Thus, attention shift between different representations is introduced, which can confound the neural activity associated with representation maintenance. In this study, we aimed to determine whether neural activity still dynamically changes during WM maintenance in the absent of any attention shift.

Debates over alpha activity are ongoing. In early times, researchers believed that alpha activity reflected an idle or inactive state of the brain, since alpha power increased when subjects closed their eyes and did nothing (Pfurtscheller et al., 1996). However, many experiments have suggested that alpha oscillations are stronger in certain cognitive tasks and play important roles in some cognitive functions, especially WM (Klimesch et al., 1999; Jensen et al., 2002; Tuladhar et al., 2007). And researchers propose the alpha functional inhibition hypothesis based on the sequential verbal Sternberg task (Jensen and Mazaheri, 2010). While, whether this hypothesis could also describe visuospatial WM (like the paradigm in this study)? Another frequency band that also plays an important role in WM maintenance is theta. Many studies have observed significant theta oscillation in WM task (for review, see Kahana et al., 2001), and its power increases along with memory load (Raghavachari et al., 2001, 2006; Jensen and Tesche, 2002; Onton et al., 2005). While, multiple stimuli are presented sequentially in these studies, i.e., sequential information presented along the time dimension. How about simultaneous information presented along the space dimension? Does the theta oscillations also elevated along with load level? Therefore, using this visuospatial WM task, the functional role of alpha and theta oscillations was also investigated.

## Materials and Methods

### Participants

Twenty-one right-handed, healthy volunteers (19–25 years old; 10 women and 11 men) participated in the EEG study. All participants had normal or corrected-to-normal visual acuity, reported normal color vision, and had no self-reported history of neurological or psychiatric disorders. This study was approved by the ethics committee of the Institute of Psychology, Chinese Academy of Sciences, and the written informed consent was obtained from each participant prior to the experiment. In accordance with the Declaration of Helsinki, the written informed consent was obtained from each subject.

### Experimental Procedure

**FIGURE 1:**
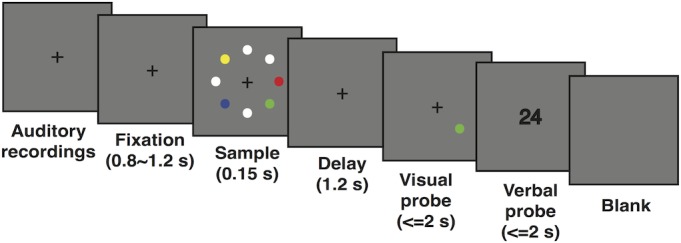
Schematic representation of the modified visuospatial delayed match-to-sample task.

All participants performed a modified visuospatial DMST (schematic representation shown in **Figure 1**). Throughout the entire trial, a cross fixation was presented at the central point to help maintain the subject’s attention. Each trial began with a double-digit sound, each digit lasting 300 ms and interleaved by another 300 ms. Subjects were instructed to keep the auditory stimuli in mind until the end of the trial. Then, the cross fixation was shown on screen for a duration randomly chosen from a uniform distribution of 800–1200 ms. The sample display was presented for a brief period of 150 ms. This very short duration effectively disabled potential eye movements. Eight dots (1.5° radius each) were presented in eight equally spaced locations along an invisible circle with an eccentricity of 5.5° from the central point. One, two, four, or seven of the eight dots were colorized randomly from eight distinguishable colors to index the level of WM load, and the rest of the dots were filled with white. Participants were clearly instructed to memorize only the colorized dots and ignore the white ones. After a delay period of 1200 ms, one colorized dot appeared as the target, and participants were asked to determine whether the color of the target dot matched that of the dot shown in the same location in the sample display. Thus, subjects needed to keep both the colors and locations of the colorized dots in mind. Finally, a visual double-digit number was presented as an auditory probe, and participants were instructed to determine whether these digits matched those played in the recording at the beginning of the trial. Therefore, the experiment contained both a visual WM task and a verbal control task. All responses were key presses and were required to be as quick (within 2 s) and accurate as possible. Each participant completed a total of 480 trials with eight conditions (4 load conditions × 2 matching conditions) randomly counterbalanced and distributed in six blocks interleaved by five break periods. Throughout the experiment, subjects were instructed to keep their heads stable, focus on the center of screen, and try their best to eliminate eye movement during the task period but not during presentation of the blank screen.

### Data Acquisition

During each trial, EEG signals were continuously recorded with 64 Ag/AgCl electrodes in a cap (Neuroscan) using the International 10–20 System with a frontocentral electrode as ground and left mastoid as reference. Data were re-referenced to a linked-mastoid (the algebraic average of the left and right mastoids) offline by a transformation matrix. In addition, horizontal and vertical electrooculograms (EOGs) were recorded at the same time from electrodes placed ∼1 cm lateral to left and right eyes, above and below the external canthi of the right eye, to measure horizontal and vertical eye movements. EEG and EOG were recorded at a sampling rate of 1000 Hz, amplified with a DC amplifier, and passed through a 0.01–250-Hz bandpass filter.

### Data Processing

Data processing was conducted using MATLAB in conjunction with EEGLAB Toolbox (van Dijk et al., 2010). Raw data were bandpass filtered from 0.01 to 100 Hz by an offline filter (Hamming windowed sinc FIR filter). An epoch (-1700 to 2800 ms) was extracted only from correct trials, aligned to the sample onset (0 ms). Any trials contaminated by big drifting and large movements were abandoned by visual inspection. Then, independent component analysis (Delorme et al., 2007) was performed to decompose the data, and a plug-in called automatic EEG artifact detector based on the joint use of spatial and temporal features (ADJUST) (Mognon et al., 2011) was applied to remove components characterized by blinks, eye movement, and muscle or cardiac artifacts. Finally, epochs were visually scanned to further exclude ones contaminated by artifacts. Participants with poor behavioral performance and numerous EEG artifacts (trial rejection rates >20%) were excluded from further analyses (4 participants—2 women and 2 men—were excluded, leaving 17 participants for further analysis).

**FIGURE 2:**
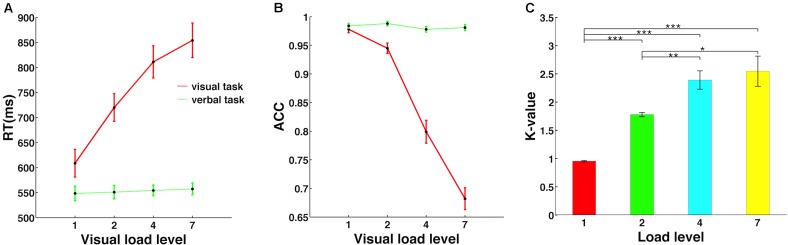
Memory load effect on behavioral performance. **(A)** The reaction time was significantly modulated by memory load level only in the visuospatial working memory task, but not in the verbal control task. **(B)** Accuracy decreased significantly with memory load increase only in the visuospatial task. **(C)** Individual memory capacity is indicated by Cowan’s *K*-value, defined as (hit rate + correct rejection rate – 1) × (memory load level). ∗, ∗∗, and ∗∗∗ mean the significance level of 0.05, 0.01, and 0.001.

For statistical analyses, analyses of variance (ANOVA) was conducted for reaction time and accuracy in different memory load conditions (**Figures 2A,B**). In order to measure individual memory capacity, Cowan’s *K*-value (Cowan, 2001), i.e. (hit rate + correct rejection rate - 1) × (memory load level), was calculated for each individual under each load condition. ANOVA was conducted to explore the change in *K*-value with load increase (**Figure 2C**). If the assumption of sphericity was violated, a Greenhouse-Geisser correction was used to adjust the degrees of freedom. Bonferroni correction was applied to a *post hoc* *t*-test to correct multiple comparisons.

The recording sites were grouped into three clusters according to their anatomical locations: frontal channel-cluster (AF3, AF4, F5, F3, F1, FZ, F2, F4, F6), central channel-cluster (central: C1, CZ, C2, CP1, CPZ, CP2; temporal: T7, C5, TP7, CP5, C6, T8, CP6, TP8; parietal: P3, P1, PZ, P2, P4) and occipital channel-cluster (PO7, PO5, PO3, POZ, PO4, PO6, PO8, CB1, O1, OZ, O2, CB2). Further analyses were all based on these three channel-clusters.

**FIGURE 3:**
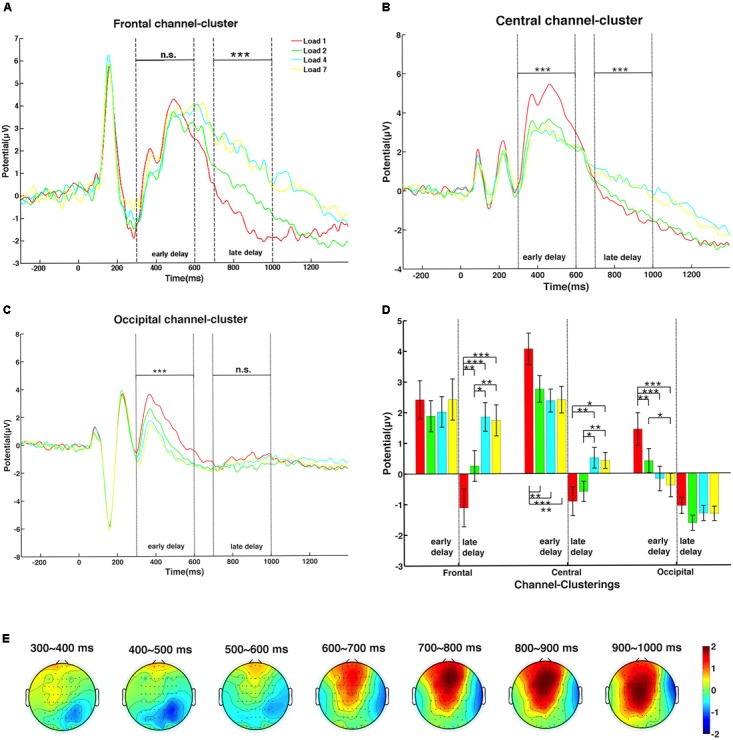
The dynamic change of the memory load effect on amplitude during the delay period. **(A)** Amplitude of the frontal channel-cluster increased significantly with memory load only in late delay. **(B)** The central channel-cluster showed significant memory load main effects, with different trends in the early and late delay stages. **(C)** The occipital channel-cluster exerted significant lower amplitude with higher memory load only in early delay. **(D)** The *post hoc* *t*-tests of amplitude in all memory load conditions of the three channel-clusters were calculated during the two delay stages (one asterisk indicates significance level of 0.05; two asterisks indicate significance level of 0.01; three asterisks indicate significance level of 0.001). **(E)** Topographic maps of mean amplitude difference between memory load levels 2 and 4 during the delay period, in steps of 100 ms.

For event-related potential (ERP) analyses, data from the three channel-clusters after preprocessing were filtered by a 30-Hz low-pass filter (Hamming windowed sinc FIR filter). Baseline correction was performed for whole epoch data from each correct trial, i.e., amplitude of each time point in the whole epoch window was subtracted from the mean amplitude of the baseline window, which was a 300-ms EEG signal before sample onset. The data obtained for the time window of -300 to 1350 ms (300 ms baseline, 150 ms sample display, and the entire 1200-ms delay duration) for each load condition were averaged across trials and then averaged across subjects to obtain the grand averaged ERPs (**Figures 3A–C**). As shown in **Figure 3**, neural activity was clearly grouped into two time clusters. Thus, the 1200-ms maintenance period was divided into early and late delay stages, 300–600 and 700–1000 ms after sample offset, respectively. ANOVAs were then performed to further explore the differences in ERPs in each delay stage (**Figure 3D**). The WM load effect on the spatial pattern of activation was plotted in topographies shown in **Figure 3E**. Because most individuals had a memory capacity between two and four objects, and the amplitude of these two conditions were significantly different, we proposed that the amplitude difference between memory load levels 2 and 4 could reflect an individual’s memory capacity. The time window for calculation was 100 ms from the beginning of early delay (300 ms after the delay onset) to the end of late delay period (1000 ms after the delay onset), stepped by 100 ms, resulting in seven sub-topographies. In each 100-ms time window, the amplitude of memory load level 2 was subtracted from level 4 point-by-point for each channel, and the amplitude differences were averaged across the time window and subjects and then projected to the topography.

**FIGURE 4:**
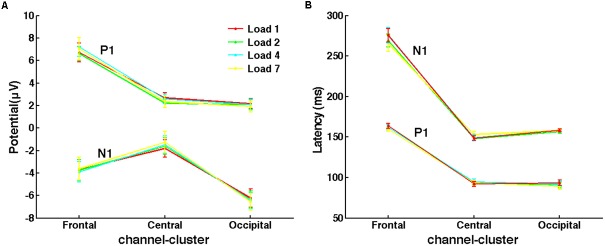
No memory load effect on evoked responses of P1 and N1 was observed. **(A)** The mean peak amplitudes of P1 and N1 under different load conditions across subjects. **(B)** The mean latency of P1 and N1 peaks.

For evoked response (ER) components, peaks of P1 and N1 were analyzed to determine whether the memory load effect emerged during the early perceptual stage. According to the grand averaged ERPs of the three channel-clusters, time windows to search the peak amplitude and corresponding latency of P1 and N1 were slightly different: 70–110 and 130–170 ms for P1 and N1, respectively, in central and occipital channel-clusters; and 130–190 and 210–320 ms in the frontal channel-cluster. For each subject and channel-cluster, peaks of these two early components in different channels were averaged under each load condition. Then, ANOVAs were performed to investigate the load effect on all three channel-clusters. The averaged P1 and N1 peaks across subjects are shown in **Figure 4**.

**FIGURE 5:**
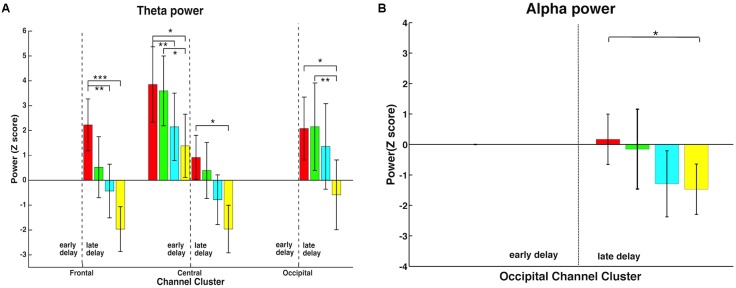
Memory load effect on time-frequency representations of theta and alpha activity. **(A)** In the late delay period, all three channel-clusters showed a significant memory load main effect in the theta band, decreasing power with increasing load. In addition, this effect was observed in early delay in the central channel-cluster. **(B)** Alpha activity also demonstrated a memory load main effect but only in the occipital channel-cluster in the late delay period, with higher memory load inducing lower alpha power. ∗, ∗∗, and ∗∗∗ mean the significance level of 0.05, 0.01, and 0.001.

Time-frequency representations were calculated by convolving the Morlet wavelets (five-cycle) with epoch data of each electrode ranging from 2 to 100 Hz in linearly increasing steps, and the -300 to 50 ms window before sample onset was used as a baseline. The 50-ms signal before the alignment point was omitted to avoid pollution of oscillatory estimates with activity from the sample period of the trial, as time-frequency representations were of poor temporal precision at low frequency. The resulting complex representations of time-frequency decomposition were squared to yield oscillatory power of each frequency point. The epoch data were normalized with respect to oscillatory power during the 250-ms baseline for each trial at each frequency. Because we focused on four frequency bands, namely theta (4–8 Hz), alpha (8–13 Hz), beta (13–30 Hz), and gamma bands (30–60 Hz), the power for frequency points within each of these four frequency bands were averaged to yield the corresponding band power estimation. Because exploratory analyses of beta and gamma power during the delay period yielded no significant results between different load conditions, further analyses focused on theta and alpha. These powers were first averaged across trials and then across subjects. ANOVAs were conducted to reveal the main effect of WM load (**Figures 5A,B**).

## Results

### Behavior Performance Parametrically Changed With Memory Load

As shown in **Figure 2**, the visuospatial WM task (red line) but not the verbal control task (green line) showed a significant main effect of memory load. Specifically, reaction time increased [*F*(3,48) = 144.69, *p* < 0.001, **Figure 2A**] and accuracy decreased [*F*(3,48) = 189.91, *p* < 0.001, **Figure 2B**] significantly with memory load increase, and *post hoc* *t*-tests revealed a significant difference for each tested load level pair. However, memory load exerted no effect on reaction time [*F*(3,48) = 0.82, *p* = 0.45] or accuracy [*F*(3,48) = 2.84, *p* = 0.07] in the verbal control task.

Cowan’s *K*-value was calculated for each memory load for each individual, and individual *K*-values were then averaged to yield the mean *K*-value, as shown in **Figure 2C**. As memory load increased, *K*-value first increased significantly and then saturated at load level 4 [*F*(3,48) = 31.58, *p* < 0.001]. Mean WM capacity across subjects was represented by the largest *K*-value in all conditions, which was 2.55 in load level 7. Therefore, this result suggested that individual WM capacity was severely limited and could maintain only about three objects in the mind at the same time, consistent with the finding of a previous functional magnetic resonance imaging (fMRI) study (Xu and Chun, 2006).

### WM Load Modulated Amplitude Dynamically in Delay Period

Event-related potentials in the three channel-clusters during the delay period were modulated by WM load, as shown in **Figure 3** (red: load 1; green: load 2; cyan: load 4; yellow: load 7). The frontal channel-cluster only showed a significant main effect of memory load in the late delay stage, with higher load level inducing a higher amplitude [early stage: *F*(3,48) = 1.38, *p* = 0.27; late stage: *F*(3,48) = 24.92, *p* < 0.001]. However, the occipital channel-cluster exerted a significant load effect only in the early delay period, with lower memory load inducing stronger neural activity [early stage: *F*(3,48) = 20.44, *p* < 0.001; late stage: *F*(3,48) = 2.48, *p* = 0.07]. In addition, the central channel-cluster showed significant load effects in both stages [early stage: *F*(3,48) = 15.29, *p* < 0.001; late stage: *F*(3,48) = 11.04, *p* < 0.001]. Interestingly, memory load effect on ERP amplitude showed clear interactions during the two delay stages. In the early stage, lower memory load induced higher amplitude, while the reverse trend was observed in the late stage. Paired comparisons of load main effect on amplitude during both delay stages are shown in **Figure 3D**.

To explicitly express the spatial dynamics of the memory load effect on neural activity changes (amplitude difference between load levels 2 and 4) at different recording sites, seven topographic maps in a 2-D circular view were displayed in steps of 100 ms during the whole delay period (for details, see section “Data Processing”). As shown in **Figure 3E**, neural activity gradually strengthened over the whole brain as time elapsed, indicating more involvement of frontal and parietal activity in the late delay stage in response to memory load increase.

### No WM Load Effect in the Perceptual Period

Several studies indicated that the limitation of WM capacity emerges at the very beginning of the delay period (Buschman et al., 2011; Palva et al., 2011). However, paradigms used in these experiments did not control the encoding load while varying the memory load. Therefore, it was unclear whether the WM limitation found in early delay resulted from encoding load. As shown in **Figure 4**, P1 and N1 peaks of different memory load conditions were almost the same in all three channel-clusters. The ANOVA of P1 and N1 peaks did not show a main effect of memory load (for peak amplitude: P1, all *p* > 0.126; N1, all *p* > 0.254; for peak latency: P1, all *p* > 0.090; N1, all *p* > 0.063). Thus, with good control of encoding load, WM limitations may occur only in the maintenance stage but not in the early perceptual stage. This supports the importance of controlling for encoding load when studying memory load in early delay and capacity limitation in visuospatial WM.

### Time-Frequency Representations in the Delay Period

In order to further explore how neural activity in different frequency bands responded to memory load changes, time-frequency analysis was performed in the early and late delay periods. Theta and alpha bands, but not beta and gamma bands, showed significant memory load effects. Therefore, further analyses focused on these two frequency bands. As shown in **Figure 5**, time-frequency representations demonstrated that memory load modulated power differentially in various channel-clusters during different delay stages, and only the significant effects are shown. In the theta band (**Figure 5A**), all three channel-clusters showed a significant decrease with memory load increase in the late delay period [frontal: *F*(3,48) = 10.17, *p* < 0.001; central: *F*(3,48) = 5.85, *p* = 0.002; occipital: *F*(3,48) = 5.45, *p* = 0.003], while in the early delay period, only the central channel-cluster expressed this significant load effect [*F*(3,48) = 7.77, *p* < 0.001]. In addition, alpha band activity showed a significant memory load main effect but only in the occipital channel-cluster during the late delay period [**Figure 5B**; *F*(3,48) = 3.40, *p* = 0.025]. Higher memory load induced lower alpha band power. Therefore, the memory load effect induced variable power changes during different delay periods.

### The Late Delay Stage Predicted Individual Performance

**FIGURE 6:**
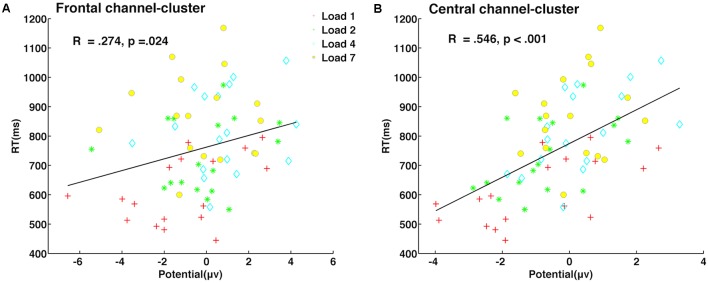
Neural activity during late delay in frontal and central channel-clusters was significantly predictive of behavioral performance. Partial correlation between neural activity amplitude and reaction time in the frontal channel-cluster **(A)** and central channel-cluster **(B)** is shown, with memory load as a covariate.

Partial correlation analyses between amplitude in both delay stages and behavior indicators (reaction time, accuracy, and capacity) were conducted, with memory load as a covariate. We found that neural activity of the late delay period in frontal (*r* = 0.274, *p* = 0.024, **Figure 6A**) and central (*r* = 0.546, *p* < 0.001, **Figure 6B**) channel-clusters was significantly and positively correlated with individual reaction time. Meanwhile, other partial correlation calculations did not reach significance. Therefore, amplitude in the frontal and central channel-clusters in the late delay period may critically contribute to visuospatial WM performance. For example, individuals with lower amplitude in the frontal and central channel-clusters during late delay perform better with lower reaction times.

## Discussion

In the present study, we found that individual visuospatial WM performance was modulated parametrically by memory load, independently of the verbal control task (**Figures 2A,B**). In addition, we did not observe significant memory load effects on early ER components after controlling for encoding load (**Figure 4**). Therefore, this modified DMST paradigm provides good control over verbal representation and encoding load confounding factors and is suitable for investigating memory load effects on visuospatial representation maintenance.

Amplitude changes of all three channel-clusters indicated that load modulation operated dynamically in the long delay period. In early delay, neural activity was more involved in visual stimuli encoding. Thus, a significant memory load effect was observed in the occipital channel-cluster. The central channel-cluster demonstrated a similar load effect. In these two channel-clusters, the simplest condition of load level one had the highest amplitude during the early delay period, perhaps because in visual stimuli encoding, the one colorized dot attracts more attention and forms an attention spotlight in contrast with the white dots. However, when neural information was transformed to higher brain regions, this effect disappeared. Notably, this effect even reversed in the late delay period. During this period, amplitude in central and frontal channel-clusters increased significantly with memory load. When WM load increased, more colorized dots were required to be maintained, resulting in greater cognitive effort reflected by the increased amplitude in higher order areas. As other studies have revealed, the effect of memory load on neural activity changes dynamically not only in the time domain, but also in the spatial domain (Cohen et al., 1997; Bashivan et al., 2014; Heinrichs-Graham and Wilson, 2015). In the present study, as memory load increased, amplitude showed temporally dynamic changes among different brain regions indicating the spread of neural activity (**Figure 3E**), which was also observed in another visuospatial WM study (Palva et al., 2011). Given that WM is a very basic process and has virtual interactions with other cognitive functions, the dynamic modulation of load during maintenance that is observed in the visuospatial task may have some implications for dual-task paradigms. For instance, WM load affects divided attention, that is, the selection of multiple objects/locations (Santangelo and Macaluso, 2013), whereas the load level does not affect visuospatial exogenous orientating (Santangelo and Spence, 2007; Santangelo et al., 2008).

Theta oscillations in rodent hippocampus are known to encode location information in the spatial domain. For example, the famous place cells (Okeefe and Recce, 1993) encode sequential information presented along the spatial dimension. Thus, theta oscillations may be sensitive to sequential information, whether spatial or temporal. When visual stimuli are presented sequentially in the time domain, theta oscillations may encode these representations one by one, resulting in power increase. However, the maintenance of simultaneously presented stimuli may be different. Some scalp EEG studies have separated the maintenance of temporal order from that of spatial location and item identity, and theta activity enhancement is found in the temporal order condition but not in spatial location or item identity conditions (Hsieh et al., 2011; Roberts et al., 2013). Another magnetoencephalography study found no significant theta power main effect during the retention period of simultaneously presented stimuli (Heinrichs-Graham and Wilson, 2015). Increased theta band neural activity was not observed in any of these visuospatial WM maintenance studies. Consistent with this, in our study, we found that theta power decreased with memory load increase. This indicates that theta oscillations are very important in the maintenance of sequential information, but are not sensitive to simultaneous information maintenance.

The hypothesis that alpha oscillations gate information through inhibition is largely based on the Sternberg task which reveals that the power of the alpha activity increases with memory load. However, in the present modified visuospatial DMST, alpha power declined in the occipital channel-cluster. Another visuospatial WM study also showed a similar alpha power decrease in occipital electrode sites (Sauseng et al., 2005). In fact, these two seemingly contradictory observations may similarly illustrate the cognitive function of alpha oscillations. In contrast to the classical Sternberg task, in which stimuli can be maintained easily in verbal form, individuals completing this visuospatial DMST are more likely to utilize a visual strategy to maintain stimuli during the delay period (e.g., keeping the sample as a visual pattern in mind). Moreover, this type of strategy is what individuals were instructed to employ to ensure purely visual representations. As a result, the occipital channel-cluster becomes a relevant region during the delay period and will be more engaged as memory load increases, reflected by a decrease in alpha power. This is consistent with the conclusion drawn from the classical Sternberg task that alpha oscillation gates neural information by functional inhibition (Klimesch et al., 2007; Jensen and Mazaheri, 2010). Results from fMRI studies also indicate that alpha increases appear in brain regions outside of the WM network (Haegens et al., 2010; Roux et al., 2012).

As mentioned above, low-frequency power decreased as memory load increased, consistent with another visual DMST study (Palva et al., 2011). Many studies have demonstrated that beta oscillations are related to the motor cortex and play an important role in human motor processing (Jensen et al., 2005; Buchholz et al., 2014). However, there was no motor activity in the maintenance of visuospatial representations during the delay period, which could explain the lack of beta oscillations observed. Gamma band oscillations are thought to have a crucial role in object perception (Tallon-Baudry and Bertrand, 1999), as well as in the maintenance of multi-object representations (Tallon-Baudry et al., 1998; Howard et al., 2003; van Vugt et al., 2010). Limited by scalp EEG recording, this study did not find significant gamma activity, perhaps because high-frequency activity is severely blurred when transformed through the skull and scalp to recording sites. Precise neural signal recording techniques, such as intracranial EEG, would be helpful to illustrate cognitive functions of high-frequency oscillations in visuospatial WM.

Undoubtedly, maintenance of multi-object representations during the entire delay period is essential for better performance. However, results from partial correlation analysis showed that neural activity in frontal and central channel-clusters during late delay was more predictive of performance. The paradigm in the present study required individuals to maintain both color information and the corresponding spatial location, which inevitably required feature binding in late delay. If the participant successfully maintained both visual color and spatial features only in early delay, while failing to perform correct feature binding in late delay, performance would be poor. Evidence from fMRI suggests that the posterior parietal cortex is a neural node of WM capacity (Todd and Marois, 2004), and neural signals from the prefrontal cortex can be used to decode contents of visual WM (Polania et al., 2012). Thus, neural activity in both central and frontal channel-clusters has an important role in maintenance and is more predictive of behavioral performance. In addition, this result is also consistent with the idea that features in the frontal cortex reflect representations that are transformed for guidance of upcoming behavioral performance (Christophel et al., 2017).

## Conclusion

In summary, during maintenance of visuospatial representations, WM load exerted contrasting effects on a distributed WM network in early and late delay periods, with late delay neural activity better predicting individual performance. In addition, alpha power in the occipital cortex decreased as memory load increased, indicating this region played a role in maintaining visuospatial representations. Thus, these findings are consistent with findings from the verbal Sternberg task suggesting that alpha oscillations gate information by inhibition.

## Author Contributions

YP and LW designed the experiment. YP and ZT were responsible for data collection and data processing. YP and LW wrote the manuscript, with the intellectual advice of ZG and YL.

## Conflict of Interest Statement

The authors declare that the research was conducted in the absence of any commercial or financial relationships that could be construed as a potential conflict of interest.
